# Behavioral and transcriptional effects of age in HbSS-BERK humanized SCD mice

**DOI:** 10.1093/jscdis/yoaf033

**Published:** 2025-10-07

**Authors:** Kennedy N Goldsborough, Michael W Taylor, Bryan D McKiver, Karan H Muchhala, Molly E Sonenklar, Atuahene Adu-Gyamfi, Sara M Herz, Dawn K Jessup, Joyce A Lloyd, Hamid I Akbarali, M Imad Damaj, Kalpna Gupta, Wally R Smith, Aron H Lichtman

**Affiliations:** Department of Pharmacology/Toxicology, Virginia Commonwealth University, Richmond, VA 23298, United States; Department of Pharmacology/Toxicology, Virginia Commonwealth University, Richmond, VA 23298, United States; Department of Pharmacology/Toxicology, Virginia Commonwealth University, Richmond, VA 23298, United States; Department of Pharmacology/Toxicology, Virginia Commonwealth University, Richmond, VA 23298, United States; Department of Pediatric Hematology/Oncology, Virginia Commonwealth University, Richmond, VA, United States; Department of Pharmacology/Toxicology, Virginia Commonwealth University, Richmond, VA 23298, United States; Department of Pharmacology/Toxicology, Virginia Commonwealth University, Richmond, VA 23298, United States; Department of Pharmacology/Toxicology, Virginia Commonwealth University, Richmond, VA 23298, United States; Department of Human and Molecular Genetics, Virginia Commonwealth University, Richmond, VA, United States; Department of Pharmacology/Toxicology, Virginia Commonwealth University, Richmond, VA 23298, United States; Department of Pharmacology/Toxicology, Virginia Commonwealth University, Richmond, VA 23298, United States; Division of Hematology/Oncology, Department of Medicine, University of California, Irvine, CA, United States; Department of Internal Medicine, Virginia Commonwealth University, Richmond, VA, United States; Department of Pharmacology/Toxicology, Virginia Commonwealth University, Richmond, VA 23298, United States

**Keywords:** SCD, HbSS Berkeley mice, pain, inflammation, opioid, oxycodone

## Abstract

**Objectives:**

SCD is associated with morbidity, mortality, and severe pain that is well modeled in humanized Berkeley SCD (HbSS) mice. Here, we conducted a comprehensive study to evaluate the effects of age on the development of the HbSS hyper-nociceptive phenotype. We also examined the antinociceptive effects of oxycodone, a commonly used analgesics to manage SCD-related pain, in both genotypes.

**Methods:**

Mixed sex, 2-, 5-, and 10-month HbSS and HbAA control mice were assessed in cadre of stimulus-evoked and non-evoked functional assays. The dose-response relationship of oxycodone was evaluated in 10-month mice from both genotypes in a subset of in vivo assays. Finally, qRT-PCR was used to quantify the relative mRNA levels of opioid receptors and ligand precursors, and pro-inflammatory cytokines, from spinal cord and dorsal root ganglia.

**Results:**

HbSS mice displayed augmented responses in stimulus-evoked assays and deficits in non-evoked functional behaviors that overall worsened in severity over age, compared with controls. Oxycodone dose-dependently attenuated mechanical hypersensitivity and produced thermal antinociception but failed to normalize (or worsened) functional behavior. Finally, HbSS mice exhibited overall or age-dependent differences in mRNA amounts of mu and kappa opioid receptors, POMC, IL-1β, and IL-6.

**Conclusions:**

This study offers a comprehensive approach to investigate candidate drugs to treat SCD pain and explores biomarkers associated with the HbSS SCD mouse model. Although oxycodone ameliorated the hyper-nociceptive phenotype of HbSS mice, it failed to restore functional behavior, underscoring the need to identify novel therapeutic strategies that effectively reduce pain and restore functional behavior.

## INTRODUCTION

SCD, a common inherited disease caused by a homozygous point mutation in the β-globin gene, results in polymerization of HbS, the characteristic sickle shape of red blood cells, and severe pain.^1^ The hallmarks of SCD (ie, hemolysis, inflammation, vaso-occlusion, vasculopathy, ischemia-reperfusion injury, organ damage, and neuropathy) contribute to chronic pain that may emerge during childhood and increase in prevalence and severity with age.^2^ By adulthood, over 55% of SCD patients experience chronic pain and 29% report pain on >95% of days^3^^,^^4^ The high prevalence of chronic pain among SCD patients and its devastating effects on quality of life underscore the need for increased understanding of the biological factors that can influence treatment.

Transgenic mice expressing human sickle Hb offer utility to investigate factors contributing to SCD chronic pain and identify novel candidate analgesics, and the homozygous Berkeley (HbSS) mouse model has many pathobiological and nociceptive features most similar to the human disease.^5–7^ Similar to SCD patients, the red blood cells of these mice become sickled, rigid and fragile, resulting in severe hemolytic anemia and ischemic injury leading to extensive multi-organ damage and a shortened lifespan.^8^ SCD mice show elevated proinflammatory markers in plasma, leukocytes, platelets, and other tissues^9–11^ as well as sensitized peripheral sensory nerve fibers^12–15^ and spinal dorsal horn nociceptors.^16^ Notably, HbSS mice display robust hypersensitive responses to mechanical, heat, and cold stimuli, as well as decreased grip strength compared to both control (HbAA-BERK) mice and the Townes SCD mouse model^7^^,^^17^ phenotypic hyper-nociceptive responses depend on the assay, age, sex, and genetic background of the mouse. Indeed, extensive characterization of HbSS mice suggests chronic hyper-nociceptive behaviors that vary with age and sex in these mice.^7^^,^^18^

Despite evidence of neuropathic pain in an estimated 25%-40% of adolescent and adult SCD patients,^19^ few studies have evaluated the underlying neuropathy that precedes the clinically evident neurological signs/symptoms. Electrophysiological and pathological alterations occur secondary to an ischemic insult during a sickling crisis, and can range from transient metabolic conduction block to segmental demyelination or Wallerian degeneration.^20^^,^^21^ Clinical and preclinical neuropathy has been histopathologically demonstrated as demyelination and axonal degradation in SCD mouse models.^7^^,^^22^ Because nerve conduction represents a quantitative method for evaluating peripheral nerve function and integrity and peripheral nerves of SCD patients show decreased conduction velocity,^23^^,^^24^ we examined nerve conductance of the caudal nerve of the tail to examine potential age-related differences in peripheral nerve function between HbSS and HbAA mice.

Here we conducted a comprehensive study to evaluate the effects of age (ie, 2, 5, and 10 months) in a battery of stimulus-evoked nociceptive (von Frey, hotplate, and acetone), non-evoked motor functional behavior (grip strength, inverted screen, wheel running, burrowing, and nesting), and nerve conductance assays in HbSS and HbAA mixed sex mice. We also tested whether sensory afferent dorsal root ganglia (DRG) neurons from HbSS mice are hyperexcitable, as a potential neural mechanism contributing to the hyper-nociceptive phenotype. Because oxycodone is among the most commonly used opioids to manage SCD-related pain,^25^ we tested whether it would ameliorate the hyper-nociceptive phenotype and behavioral functional deficits in 10-month HbSS mice. Finally, we used quantitative real-time polymerase chain reaction (qRT-PCR) to examine whether HbSS mice show alterations in relative mRNA levels of components of the endogenous opioid system as well as pro-inflammatory cytokines.

## MATERIALS AND METHODS

### Animals

All animal procedures were performed in accordance with the guidelines of the National Institutes of Health (NIH) and were ethically reviewed and approved by the Institutional Animal Care and Use Committee at Virginia Commonwealth University. Adult male and female HbSS-BERK and HbAA-BERK mice bred in the VCU Transgenic/Knockout Mouse Core. Separate cohorts of mice at 2, 5, and 10 months of age (all ages are designated within ± 2 weeks) were assessed at the same time point. While genotype and age represented primary independent variables and were adequately powered; sex was a secondary variable and though equal numbers of male and female mice were included for each experiment, this factor was underpowered (see Ref. 26 for explanation). BERK mice lack murine alpha- and beta-globin genes, and express human alpha-, and beta-globin. HbSS mice have the human beta-globin gene with the SCA mutation, and HbAA mice express normal human HbA and served as controls. Both HbAA and HbSS mice are on a highly mixed background consisting of the same original strain contributions (FVB/N, 129, DBA/2, Black Swiss, and >50% C57BL/6) and comparable genetic variability within each group. For oxycodone experiments, 10-month-old mice (both sexes) were used because they most robustly expressed the chronic hyper-nociceptive phenotype and motor functional deficits in all assays. One to five mice were housed per cage, with group housed mice consisting of same sex littermates. The vivarium consisted of a temperature- (20-22°C), humidity- (55 ± 10%), and light-controlled (12-hour light/dark; lights on at 0600) in an AAALAC-approved facility, with standard rodent chow and water available ad libitum.

### Drugs

Oxycodone (Sigma-Aldrich, St Louis, MO) was dissolved in a saline (0.9% NaCl; sterile) solution and was administered via subcutaneous (s.c.) injection in a volume of 0.1 mL/10 g of body weight, which yielded doses of 0, 1.25, 2.5, 5, and 10 mg/kg.

### Mechanical hypersensitivity

The up-and-down method in von Frey test was used to determine paw withdrawal thresholds (PWT) to non-noxious mechanical stimuli.^27^ For acclimation to the test environment, mice were unrestrained, placed under individual inverted ventilated acrylic cylinders on a wire mesh screen (spaces 0.5 mm apart), and covered with a dark sheet. Mice were habituated for approximately 30 min/day for two days prior to testing. The von Frey test utilizes a series of calibrated monofilaments (2.83-4.31 log stimulus intensity; North Coast Medical, Morgan Hills, CA) applied randomly to the left and right plantar surface of the hind paw for 3 s. Lifting, licking, or shaking the paw in response to the filament was considered a response. PWT from both hind paws were averaged for each mouse.

### Thermal nociception

The hotplate test was used to evaluate sensitivity to a noxious heat stimulus.^28^ The mice were individually placed on a hotplate maintained at 52.0 ± 0.2°C (Columbus Instruments, Columbus, OH, USA). The latency to the first paw response (lifting, licking, and shaking the paw) was recorded with a 20 s cut-off time to avoid tissue damage.

### Cold hypersensitivity

The acetone test was used to evaluate cold hypersensitivity.^29^ Mice were placed in ventilated acrylic cylinders on a wire mesh screen (spaces 0.5 mm apart) and covered with a dark sheet for 30 min prior to testing. A micropipette was used to dispense 10 uL of acetone (Sigma-Aldrich, MO, USA) onto the plantar surface of each hind paw. Time spent licking or shaking the hind paw was recorded during a 60 s observation period. Time spent responding (s) was averaged for both paws.

### Grip strength

The grip strength test was used to assess motor function, muscular strength, and musculoskeletal/deep tissue hyperalgesia.^30^ The subject’s forepaws were gently placed on a horizontal metal bar until it gripped the bar, while the experimenter gently held the middle portion of the mouse’s tail. The experimenter then gently pulled on the tail to extend the mouse. The maximal grams of force the mouse applied while gripping a bar connected to a sensor (Grip Strength Meter, Ugo Basile (Thermo-Fisher), Gemonio VA, Italy) was measured for five trials carried out in succession with a 5-10 s inter-trial interval. For each mouse, the mean of the five trials were determined.

### Inverted screen

The inverted screen test requires muscle strength of all four limbs with a component of motivation as well.^31^ The apparatus used was 43 cm x 43 cm wire mesh consisting of 12 mm squares of 1 mm diameter wire and bordered by a 4 cm deep metal edge to prevent climbing to the other side. The mouse was placed in the center of the wire mesh, the timer started, the mesh flipped over and held approximately one foot above a flat surface. The timer was stopped when the mouse fell or 120 s elapsed.

### Wheel running

Wheel Running was used to assess voluntary activity as measured by the total number of wheel rotations.^32^^,^^33^ Mice were placed in wheels (Rat Activity Wheel, Lafayette Instrument Co., Lafayette, IN) with a diameter of 36 cm and a width of 11 cm. An electronic sensor recorded the number of complete rotations of the wheel during a 2-hour session.

### Burrowing

The burrowing assay is a preclinical rodent model that is sensitive to pathologies that result in hyper-nociceptive responses.^34^ Mice were acclimatized to a specific burrowing tube for two consecutive days before the burrowing the test day.^35^ Pairs of subjects were placed into empty cages for 1 hour of habituation. The burrows were fabricated in-house at VCU and consisted of hollow plastic tubes (320 mm long × 100 mm diameter) sealed at one end and open at the other with the open entrance raised approximately 60 mm above the ground to prevent loss of gravel when the burrow was placed on the floor of the test cage. Each burrow was filled with 100 g of gravel, placed into the test cage, and the test period was 1 hour in duration. The procedure was repeated on the second training day. If on the first training day, a pair of mice did not show burrowing, then a social facilitation procedure was used. Specifically, one mouse of this pair was swapped with a littermate mouse from a pair that displayed burrowing, prior to the second training session. All pairs of mice burrowed (>10%) following social facilitation; thus, none were excluded. On the test day (the day following the second baseline session), mice were individually placed into empty cages (same as used for training) for a period of habituation. A burrow filled with 100 g of corn cob bedding was placed into the cage and the mice were allowed to burrow for 1 hour. At the conclusion of the test period, the remaining gravel in the burrow was weighed and the amount of the displaced gravel calculated. The burrowing activity (% bedding removed) was calculated by subtracting the weight of bedding present at the end of the experiment from the starting weight (100 g) and expressing the proportion of bedding that had been displaced as a percentage.

### Nesting

Nesting is an innate behavior in mice that is sensitive to noxious stimuli and responsive to analgesics.^36^ Prior to experimentation, mice are individually housed with cardboard tubes for enrichment for at least one week to acclimate and assess baseline nesting behavior. Experimental controls that nested during the initial acclimation were included in the study. As we expect nesting behavior to be disrupted in sickle mice, all HbSS mice were included. Twenty-four hours prior to the start of the experiment, mice were placed in clean cages. On the test day, old nestlet material and cardboard tubes were removed, 2.54 cm × 2.54 cm nestlet squares were placed in the center of the opposing short walls of the cage (at a distance of 23 cm apart), and the mouse was returned to its cage and the experimenter left the room. After a 90 min nesting test session, the cage top was removed, the distance between the center of mass for each nestlet was measured to the nearest quarter inch. Nestlet consolidation was represented as % maximum possible effect = (change in distance/initial distance) * 100 [9,10].

### Sensory nerve conduction studies

The measurement of caudal nerve conduction was conducted as previously described.^37^ Mice were anesthetized using a constant stream of 2.5% isoflurane in oxygen using a nose cone and vaporizer (VetEquip Inc, Pleasanton, CA, USA). The dorsal caudal nerve conduction was recorded in the tail with needle electrodes and the electrophysiological recording device PowerLab/26T (ADInstruments, Colorado Springs, USA). The recording needle electrodes were placed at the base of the tail at 0.5 cm. Stimulating needle electrodes were placed at the end of the tail at 5 cm. The ground electrode was placed between the two at 2.5 cm. The sensory nerve conduction was measured after stimulation with a supramaximal impulse of 0.1 ms and 5 Hz and intensities of 8 mA and the corresponding amplitudes were recorded.

### Primary culture of dorsal root ganglia neurons and whole-cell patch-clamp electrophysiology

Primary cultures of dorsal root ganglia (DRG) neurons were prepared from 5-month-old adult female HbSS and HbAA mice as previously described.^38^ HbSS mice of this age exhibited phenotypes in multiple measures (eg, hot-plate, grip strength, inverted screen, wheel running, burrowing, sensory nerve conduction), which were not affected by sex. Briefly, intact lumbar L3-sacral S1 DRGs were harvested and enzymatically digested to obtain single cells. Dissociated DRG cells were plated on glass coverslips pre-coated with 100 μg/mL poly-d-lysine and 50 μg/mL laminin (MilliporeSigma, Burlington, MA) and maintained in Neurobasal A media supplemented with 1% FBS (Gibco, Waltham, MA), 1× B-27 (Gibco, Waltham, MA), 10 ng/ml glial line-derived neurotrophic factor (Neuromics, Edina, MN), 2 mM GlutaMax (Gibco, Waltham, MA), and 100 U/ml penicillin/streptomycin/amphotericin B (Corning, NY) at 37°C in a humidified 5% CO_2_/air stabilized incubator.

Whole-cell patch clamp electrophysiology in the current-clamp mode was used to evaluate the excitability of isolated DRG neurons 15-18 hours following dissociation as described previously.^38^ The external solution contained 135 mM NaCl, 5.4 mM KCl, 0.33 mM NaH_2_PO_4_, 5 mM HEPES, 1 mM MgCl_2_, 2 mM CaCl_2_, and 5 mM glucose (pH adjusted to 7.4 with 1 M NaOH), and the internal pipette solution contained 100 mM L-aspartic acid (K salt), 30 mM KCl, 4.5 mM Na_2_ATP, 1 mM MgCl_2_, 10 mM HEPES, 0.1 mM EGTA, and 0.5 mM NaGTP (pH adjusted to 7.2 with 3 M KOH). Current-clamp recordings were obtained at room temperature using the HEKA EPC-10 amplifier (HEKA, Bellmore, NY) from small-diameter DRG neurons (cell capacitance < 30 pF) because previous studies indicated that these neurons comprise Aδ and C-fiber nociceptors.^39–41^ A 150-millisecond pulse was applied in 10-pA steps increasing from −30 pA. Neuronal excitability included the following measures: (1) Threshold current required to elicit the first action potential, denoted as rheobase; (2) the number of action potentials evoked at two-times rheobase (2 X rheobase); (3) Resting membrane potential in the absence of holding current, (4) action potential threshold identified as the membrane voltage at which the first derivative of the action potential (dV/dt) exceeded 5 mV/ms; (5) action potential height; (6) cell capacitance, and (7) action potential half-width.

### Evaluation of oxycodone in a subset of assays

Oxycodone (0, 1.25, 2.5, 5, 10 mg/kg) was administered via s.c. injection to 10-month-old HbSS- and HbAA-mixed sex mice (N = 8/genotype) 30 min prior to testing. Doses of oxycodone were chosen based on published studies examining antinociceptive effects in acute and tail-flick and hotplate tests.^42^ A pretreatment time of 1 h was selected based on pilot time-course experiment (data not shown). These experiments were performed as a within-subject, Latin-square design with a 72-hour washout period between test days.

The antinociceptive effects of oxycodone were assessed in a subset of stimulus-evoked nociceptive assays as well as functional behavioral assays to optimize throughput. The von Frey and hotplate tests were conducted during the same test sessions, followed by evaluation in separate test sessions consisting of grip strength and inverted screen assays. For the nesting assay, we tested 2.5 and 5 mg/kg of s.c. oxycodone, because these doses produced statistically significant anti-allodynic effects only in HbSS mice.

### mRNA amounts of components of the endogenous opioid system and pro-inflammatory cytokines in mouse DRG and spinal cord

Naïve 5-month-old (HbAA: *n* = 6 male/6 female, HbSS: *n* = 2 male/7 female) and 10-month-old (HbAA: *n* = 4 male/5 female, HbSS: *n* = 2 male/7 female) mice were euthanized via rapid decapitation. At the time these experiments were conducted, the breeding colonies had been terminated; thus, tissue from 2-month-old mice was not available. The section of the spinal cord overlaying the T13-L1 vertebrae and L1-6 lumbar DRG were collected, immediately snap frozen in liquid nitrogen, and stored in a −80°C freezer. The Invitrogen PureLink RNA Mini Kit (Cat#12183025, Thermo Fisher scientific, Waltham, MA, USA) was used to isolate purified RNA from homogenized tissue samples, which were immediately quantified using the QuBit 3.0 fluorimeter (Thermo Fisher scientific, Waltham, MA, USA). Samples was diluted to 20 ng/µl RNA concentrations and were used to generate cDNA via the iScript cDNA Synthesis Kit (Biorad, Hercules, CA, USA). Quantitative real time polymerase chain reaction (qRT-PCR) was used to quantify mRNA amounts of OPRM1 (Mm01188089_m1 Oprm1, ThermoFisher scientific), OPRK1 (Mm01230885_m1 Oprk1, ThermoFisher Scientific), OPRD1 (Mm01180757_m1 Oprd1, Thermo Fisher Scientific), POMC (Mm0435874_m1 Pomc, Thermo Fisher Scientific), PDYN (Mm00457573_m1 Pdyn, Thermo Fisher Scientific), PENK (Mm01212875_m1 Penk, Thermo Fisher Scientific), IL-1β (Mm00434228_m1 Il1b, Thermo Fisher scientific), IL-6 (Mm00446190_m1 Il6, Thermo Fisher Scientific), and TNFα (Mm00443258_m1 Tnf, Thermo Fisher Scientific) in lumbar DRG and spinal cord samples. Glyceraldehyde-3-Phosphate Dehydrogenase (GAPDH) (Mm99999915_g1 Gapdh, Thermo Fisher Scientific) was used as a reference gene for all samples. All qRT-PCR assays were performed using the TaqMan Gene Expression Master Mix (Thermo Fisher Scientific, Waltham, MA, USA), loaded on 384-well plates (Biorad, Hercules, CA, USA), and ran on a QuantStudio 5 Real-Time PCR System (Thermo Fisher Scientific, Waltham, MA, USA). The fold change in gene expression (2ΔΔCT) was determined by normalizing all groups to 5-month HbAA mice.

### Statistical analyses

Two-way analysis of variance (ANOVA) was used to analyze the factors of genotype X age on each dependent measure, followed by Tukey’s post-hoc test. Three-way ANOVAs that included these independent variables and the secondary, under-powered factor of sex were also conducted.^26^ In the electrophysiology DRG experiment, two-group comparisons were performed via two-tailed unpaired Student’s t-tests. “N” represents the total number of animals, and “n” the total number of cells. For oxycodone studies, two-way repeated measures ANOVAs were conducted (genotype × dose), followed by Tukey’s post-hoc test or Bonferroni planned comparisons. Ordinary two-way ANOVA and Tukey’s post hoc analysis were used to determine differences in amounts of mRNA quantified between experimental groups. Sex differences could not be determined due to lower number of HbSS male mice, particularly in the 10-month group.

For all data, a *P* value of <.05 was considered statistically significant. The computer program GraphPad Prism version 8 (GraphPad Software Inc., San Diego, CA) was used in all statistical analyses. Data are plotted in scatter plots with mean ± standard deviation (SD).

## RESULTS

### HbSS-BERK mice exhibit a progressive hyper-nociceptive phenotype

**Figure 1.**
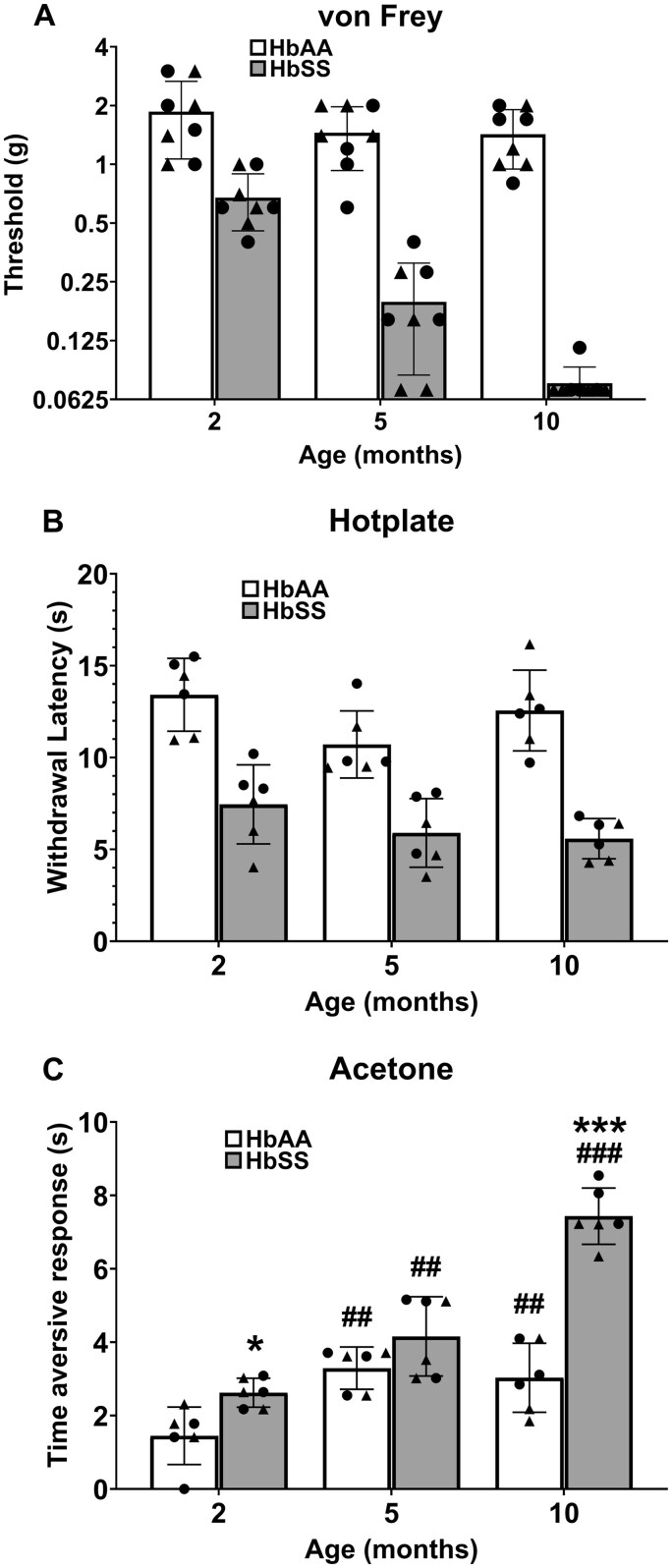
HbSS mice exhibit an age-dependent hyper-nociceptive phenotype. Separate groups of 2-, 5-, and 10-month-old, male (triangles) and female (circles) HbSS and HbAA mice were assessed for mechanical hypersensitivity (von Frey filaments), thermal hyperalgesia (hotplate test), and cold hypersensitivity (acetone-induced paw flinching). HbSS mice displayed significant mechanical hypersensitivity (Panel A) and thermal hyperalgesia (Panel B) compared with HbAA mice irrespective of age. Two-way ANOVA revealed significant main effects of genotype and age, but no genotype × age interaction for both measures (see Table S1). (Panel C) A cold allodynic phenotype emerged in the HbSS mice at 10 months, as indicated by a significant genotype × age interaction for acetone-induced paw flinching (see Table S1). * *P* < .05; ** *P* < .01; *** *P* < .001 for HbSS vs HbAA mice. # *P*  < .05; ## *P*  < .01; ### *P*  < .001 for 5- or 10-months vs 2 months. Bar graphs reflect mean ± SD from 6 to 8 mice per genotype/age. See Table S1 for two-way ANOVA for each dependent measure.

Figure 1 depicts stimulus-evoked nociceptive responses in 2-, 5-, and 10-month-old HbSS and HbAA mice (see Table S1 for two-way ANOVAs). HbSS mice exhibited mechanical hypersensitivity compared to HbAA mice, regardless of age (Figure 1A). The analysis also revealed a significant main effect of age, which was driven by 5- and 10-month mice showing increased sensitivity to the von Frey filaments compared with 2-month mice, regardless of genotype. Similarly, significant main effects of genotype and age were found in the hotplate assay, which reflect that HbSS mice exhibited thermal hyperalgesia compared with HbAA mice and thermal nociceptive responses increased over age (ie, decreased latencies in 5-month mice), regardless of genotype (Figure 1B). As shown in Figure 1C, ANOVA revealed a significant age × genotype interaction in the acetone test, resulting from significant allodynia in 10-month HbSS mice compared with HbAA mice of the same age. In addition, 5- and 10-month-old mice showed increased responsivity to the acetone flinching test compared to 2-month-old mice.

### HbSS-BERK mice exhibit significant impairment in non-evoked motor functional behaviors

**Figure 2.**
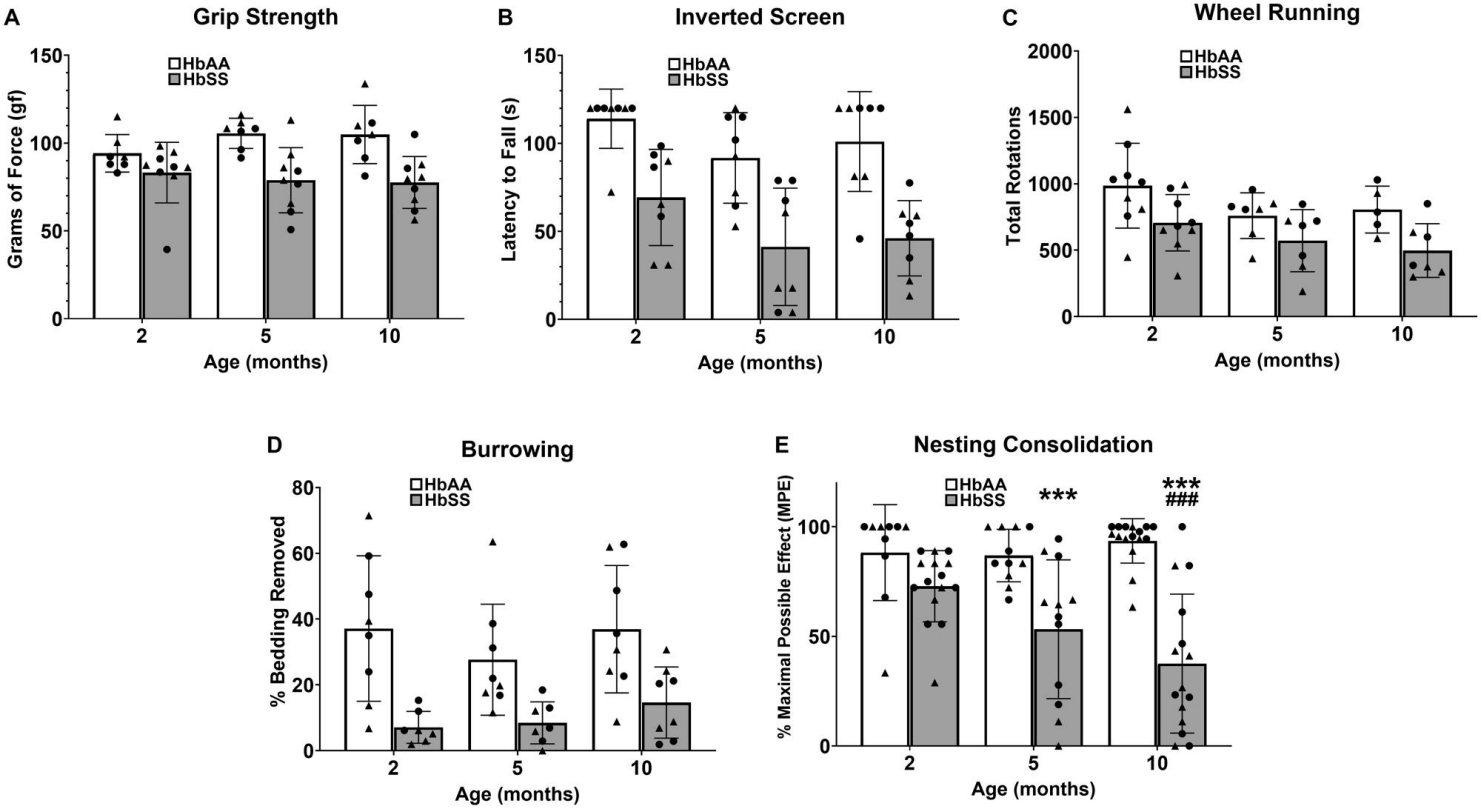
HbSS mice exhibit significant impairment in motor functional behaviors. Separate groups of HbSS and HbAA mice at 2-, 5-, and 10-months were assessed for motor function behavior. HbSS mice showed significant reductions in grip strength (Panel A), latency to fall from the inverted screen (Panel B), wheel running (Panel C), and burrowing behavior (Panel D) compared to HbAA mice. Also, irrespective of genotype, 5-month and 10-month mice exhibited worsened inverted screen performance and wheel running behavior compared to 2-month mice. Data for Panels A-D are shown as mean ± SEM from 7-9 mice per genotype/age. (Panel E) HbSS mice exhibited deficits in nestlet consolidation at 5 and 10 months compared with HbAA mice (significant genotype x age interaction). Scatter plot symbols: Triangles (males) and Circles (females); * *P* < .05; ** *P* < .01; *** *P* < .001 in age-matched HbSS mice vs HbAA mice. # *P* < .05; ## *P* < .01; ### *P* < .001 for 5/10 months vs 2 months. Bar graphs reflect mean ± SD from 13 to 16 mice per genotype/age. See Table S2 for two-way ANOVA for each dependent measure.

**Figure 3.**
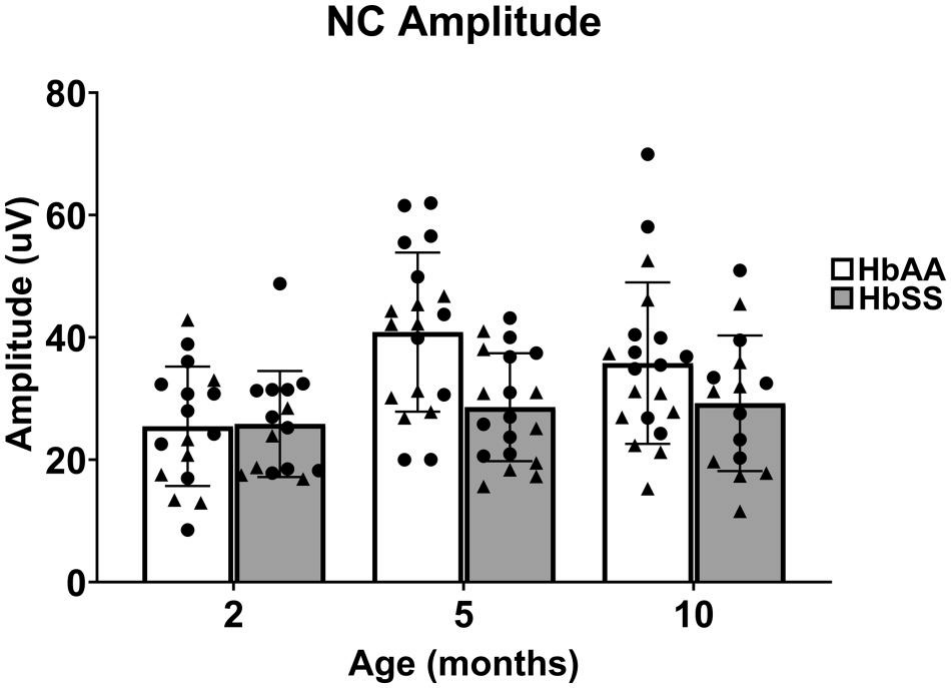
HbSS mice exhibit decreased caudal tail nerve amplitudes. Separate groups of 2-, 5-, and 10-month-old HbSS and HbAA mice were assessed for sensorimotor function of the caudal tail nerve. HbSS mice showed a significant reduction in amplitude compared with HbAA mice irrespective of age. In addition, older mice had significantly larger amplitudes than 2-month-old mice irrespective of genotype. Scatter plot symbols: Triangles (males) and Circles (females); Data are depicted as mean ± SD, with 17-20 mice per genotype/age. See Table S3 for two-way ANOVA.

HbSS mice displayed worse performance than HbAA mice in each of the five non-evoked motor functional behavioral assay, which included grip strength (Figure 2A), inverted screen (Figure 2B), wheel running (Figure 2C), burrowing (Figure 2D), and nesting (Figure 3E) assays (see Table S2 for two-way ANOVAs). Additionally, significant effects of age were found for inverted screen and burrowing, regardless of age. Lastly, a significant age × genotype interaction for nesting consolidation was found, in which HbSS mice exhibited deficits at 5 and 10 months compared with HbAA mice (Tukey post hoc analysis). Overall, these data show that HbSS mice possess a phenotypic impairment in non-evoked motor functional behaviors, which in the case of nesting behavior worsened with age.

### HbSS-BERK mice exhibit decreased amplitude of the caudal tail nerve

The effects of genotype and age on caudal nerve compound action potential amplitude are shown in Figure 3. A significant main effect was found for genotype (F (1, 99) = 8.08; *P* < .01) in which amplitude was reduced in HbSS mice compared to HbAA mice. In addition, a significant main effect was found for age (F (2, 99) = 6.22, *P* < 0.01), which resulted from increased amplitudes in 5- and 10-month-old mice compared to 2-month-old mice. The genotype × age interaction failed to achieve statistical significance (F (2, 99) = 2.85; *P* = 0.063), which precluded further post hoc analyses.

### Assessment of sex effects in stimulus-evoked nociceptive responses, non-evoked motor functional behaviors, and peripheral nerve conductance

Three-way ANOVAs were conducted to assess potential sex effects, which was underpowered and represented a secondary measure. No main effect of sex or interactions with sex were found for the von Frey, acetone-induced flinching, and burrowing assays. However, a main effect of sex was found in the hotplate assay (F (1,24) = 5.37, *P* < 0.05) in which female mice had reduced latencies compared to male mice. Additionally, female mice showed diminished grip strength compared to male mice (F (1,36) = 8.82, *P* < 0.01), though when the data were normalized for body mass, the transformed data showed higher values in females than in males. Main effects of sex were also found in the inverted screen (F (1, 36) = 5.60, *P* < 0.05) and wheel running (F (1, 32) = 5.12, *P* < 0.05) assays, with females showing improved performance compared to males, regardless of other factors. For nerve conductance, female mice also showed increased nerve conductance amplitude compared to male mice irrespective of genotype and age (F (1, 93) = 8.05, *P* < 0.01). In sum, no significant interactions were found between sex and genotype or age in the nine assays conducted.

### HbSS-BERK mice possess hyperexcitable primary afferent dorsal root ganglia neurons

**Figure 4.**
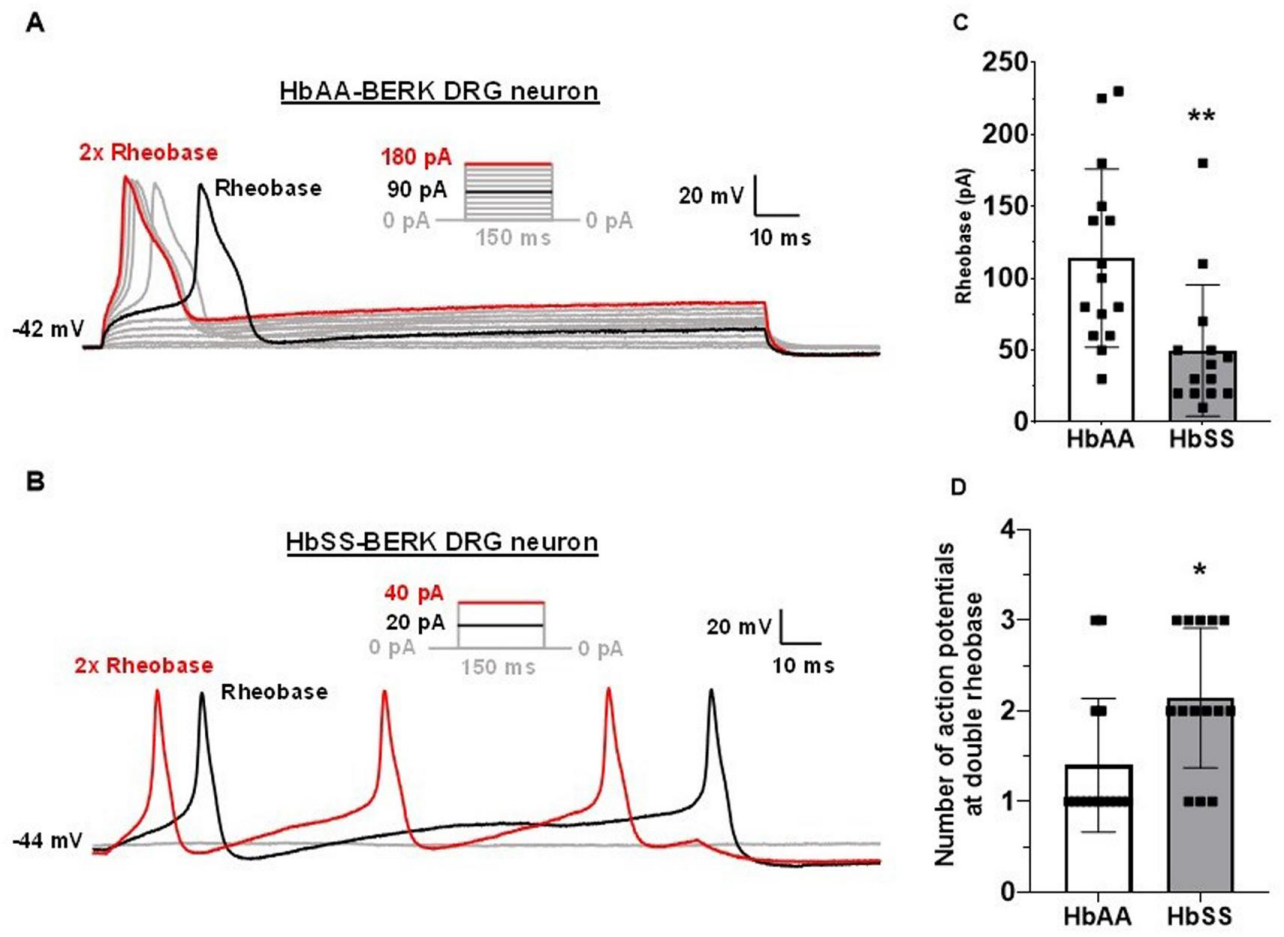
HbSS mice possess highly excitable primary afferent dorsal root ganglia neurons. Representative whole-cell patch clamp recordings of a DRG neuron isolated from a 5-month-old female HbAA mouse (Panel A) or a 5-month-old female HbSS mouse (Panel B) showing action potentials at Rheobase (ie, threshold stimulus required to elicit an action potential; black) and 2x Rheobase elicited by a 150 msec current stimulus. Corresponding step-protocol indicates Rheobase and 2x Rheobase values in pA. (Panel C) DRG neurons from HbSS mice showed a significant reduction in Rheobase compared with DRG neurons from HbAA mice. Reduced Rheobase values in HbSS mouse DRG neurons indicate increased neuronal hyperexcitability. (Panel D) 2x Rheobase stimulation elicits significantly more action potentials in DRG neurons from HbSS mice than from HbAA mice Recordings were from 14 neurons from four 5-month-old female HbSS mice and 15 neurons from four 5-month-old female HbAA mice. Data reflect mean ± SD depicted in Panels C and D. Data were analyzed using two-tailed unpaired Student’s t-tests.

As DRG neurons have been implicated in the development of chronic neuropathic pain,^43^ we used whole-cell patch clamp electrophysiology to test excitability of small diameter sensory DRG neurons in 5-month-old female HbSS mice and HbAA mice. As shown in Figure 4A-C, HbSS mouse DRG neurons exhibited reduced threshold rheobase stimulation compared to HbAA mouse DRG neurons (50 ± 12 pA versus 114 ± 16 pA; t (df) = 3.165 (27), *P* < .01). Furthermore, DRG neurons from HbSS mice showed significantly more evoked action potentials than those from HbAA mice (2.1 ± 0.21 action potentials versus 1.4 ± 0.19 action potentials; t (df) = 2.65 (27), *P* < .05; Figure 4D) at 2× rheobase. The resting membrane potential, cell capacitance, action potential threshold, action potential height, and action potential half-width of DRG neurons did not significantly differ between the genotypes. The resting membrane potentials of neurons recorded from HbAA and HbSS mice were −49.6 ± 2.2 mV and −48.2 ± 1.4 mV, respectively [t (df) = 0.525 (27), *P* = 0.60]. The capacitances of DRG neurons from HbAA and HbSS mice were 22.5 ± 1.7 pF and 18.5 ± 1.9 pF, respectively [t (df) = 1.56 (27), *P* = .13]. The mean action potential thresholds for HbAA and HbSS neurons were −17.4 ± 1.7 mV and −13.5 ± 1.5 mV, respectively [t (df) = 1.694 (27), *P* = .10]. The mean action potential heights for HbAA and HbSS neurons were 67.8 ± 3.4 mV and 61.1 ± 4.3 mV, respectively [t (df) = 1.245 (27), *P* = 0.22]. The mean action potential half-width values for HbAA and HbSS neurons were 4.4 ± 0.4 mV and 3.6 ± 0.3 ms, respectively [t (df) = 1.524 (27), *P* = 0.14]. These data indicate that small-diameter DRG neurons from HbSS female mice exhibit enhanced excitability compared to those from comparable HbAA mice at 5 months.

### Oxycodone elicits differential efficacy in a subset of stimulus-evoked and non-evoked *in vivo* assays

**Figure 5.**
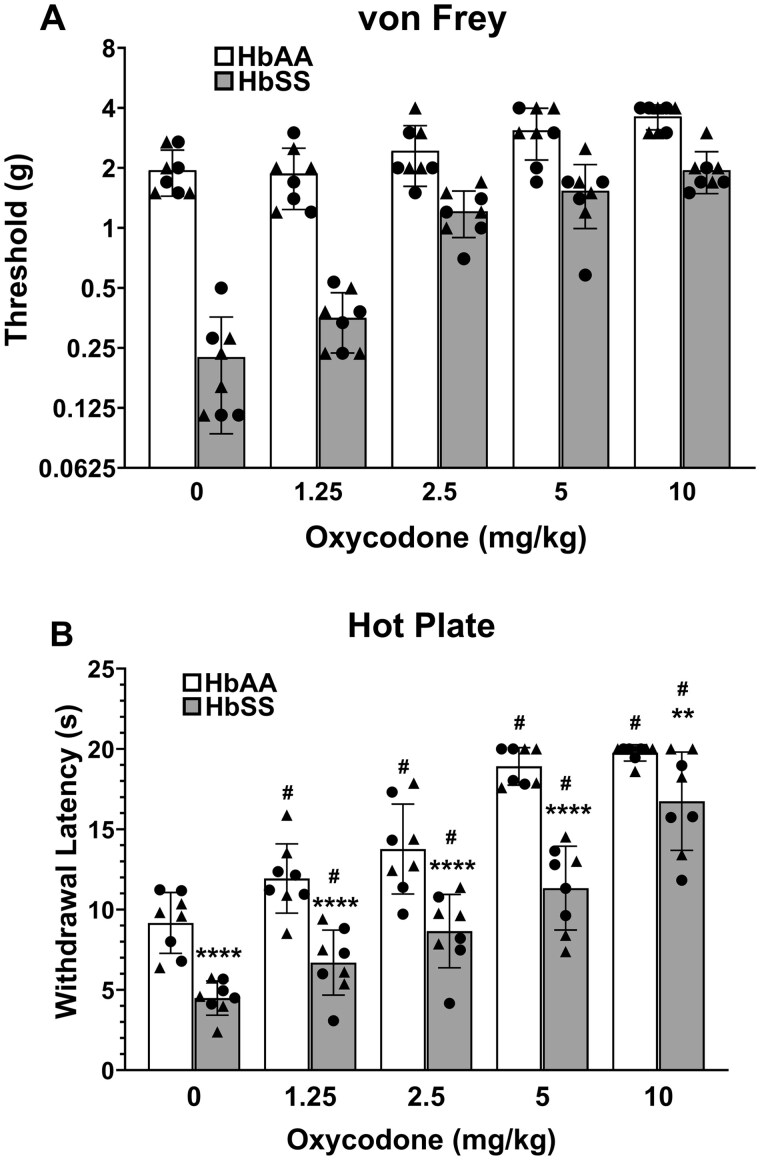
Oxycodone dose-dependently produces mechanical and thermal antinociception in HbSS mice. (Panel A) Oxycodone dose-dependently reversed mechanical hypersensitivity in HbSS mice, with only a modest increase in withdrawal thresholds in HbAA mice at the highest dose (10 mg/kg). (Panel B) Oxycodone produced dose-dependent thermal antinociception in HbSS mice, with the highest dose exceeding basal hotplate latencies from HbAA mice. Oxycodone also produced thermal antinociception in HbAA mice at all doses. Bar graphs reflect mean ± SD from 8 mice/genotype. * *P* < .05; ** *P* < .01; *** *P* < .001 in age-matched HbSS mice vs HbAA mice. # *P* < .05; ## *P* < .01; ### *P* < .001 for any dose vehicle. Scatter plot symbols: Triangles (males) and Circles (females). See Table S4 for two-way ANOVA for each dependent measure.

Having established that 10-month-old HbSS mice display the most severe overall hyper-nociceptive phenotype, we used mice of this age to test whether oxycodone would normalize a subset hyper-nociceptive responses and functional behavioral deficits in HbSS mice vs. HbAA mice (see Table S4 for two-way ANOVAs.). Oxycodone dose-dependently elevated von Frey thresholds regardless of genotype (Figure 5A). In the hot plate assay, a significant dose X genotype interaction was found, in which vehicle-treated HbSS mice showed hyper-nociceptive responses compared to vehicle-treated HbAA mice, but oxycodone dose-dependently produced thermal antinociception in both genotypes (Figure 5B).

**Figure 6.**
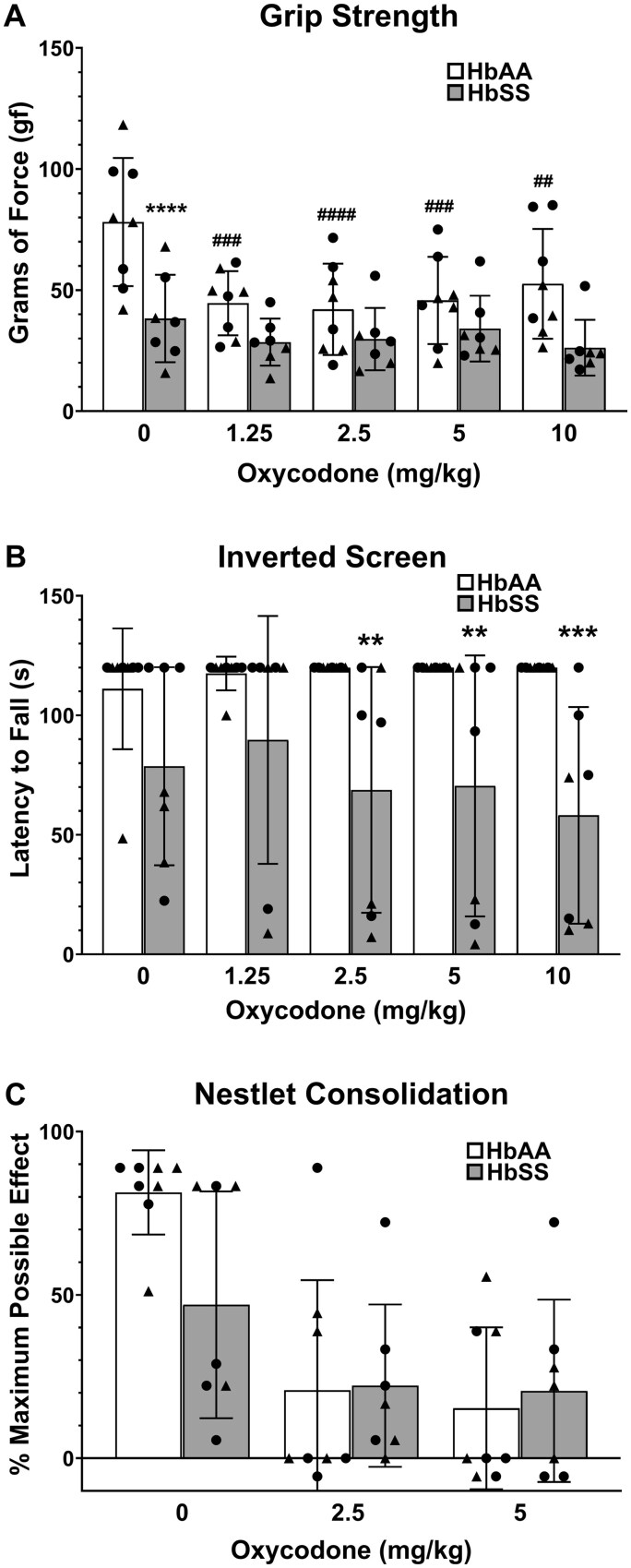
Oxycodone disrupts non-evoked functional behavior in HbSS mice. (Panel A) HbSS mice showed a significant reduction in grip strength compared with HbAA mice. While oxycodone significantly reduced grip strength in HbAA, it did not produce further grip strength deficits in HbSS mice (significant genotype × drug interaction). (Panel B) Oxycodone reduced latency to fall in HbSS, but not in HbAA mice (significant genotype X drug interaction). (Panel C) Oxycodone disrupted nesting behavior (nestlet consolidation) irrespective of genotype. Bar graphs reflect mean ± SD from 8 mice/genotype. * *P* < .05; ** *P* < .01; *** *P* < .001 in age-matched HbSS mice vs HbAA mice. # *P* < .05; ## *P* < .01; ### *P* < .001 for any dose vs. 0 mg/kg. Scatter plot symbols: Triangles (males) and Circles (females). See Table S4 for two-way ANOVA for each dependent measure.

In contrast to the stimulus-evoked assays, oxycodone had no effect or worsened performance in grip strength, inverted screen, and nesting assays (Figure 6). Significant dose × genotype interactions were found in grip strength (Figure 6A) and inverted screen (Figure 6B). Oxycodone significantly reduced grip strength in HbAA mice at 1.25 mg/kg and higher doses, but did not affect low grip strength in HbSS mice. Conversely, oxycodone impaired performance in the inverted screen test in HbSS mice, but not in HbAA mice. Finally, oxycodone significantly worsened nestlet consolidation scores in both genotypes (Figure 6C).

### mRNA amounts change for components of the endogenous opioid system and pro-inflammatory cytokines in spinal cord and DRG

In the final series of experiments, we used qRT-PCR to test whether genotype and age (5 vs. 10 months) affect relative mRNA amounts for components of the endogenous opioid system and pro-inflammatory cytokines in DRG and spinal cord. The statistical analyses (ie, two-way ANOVAs) for mRNA changes in components of the endogenous system and pro-inflammatory cytokines are presented in Tables S5 and S6, respectively.

**Figure 7.**
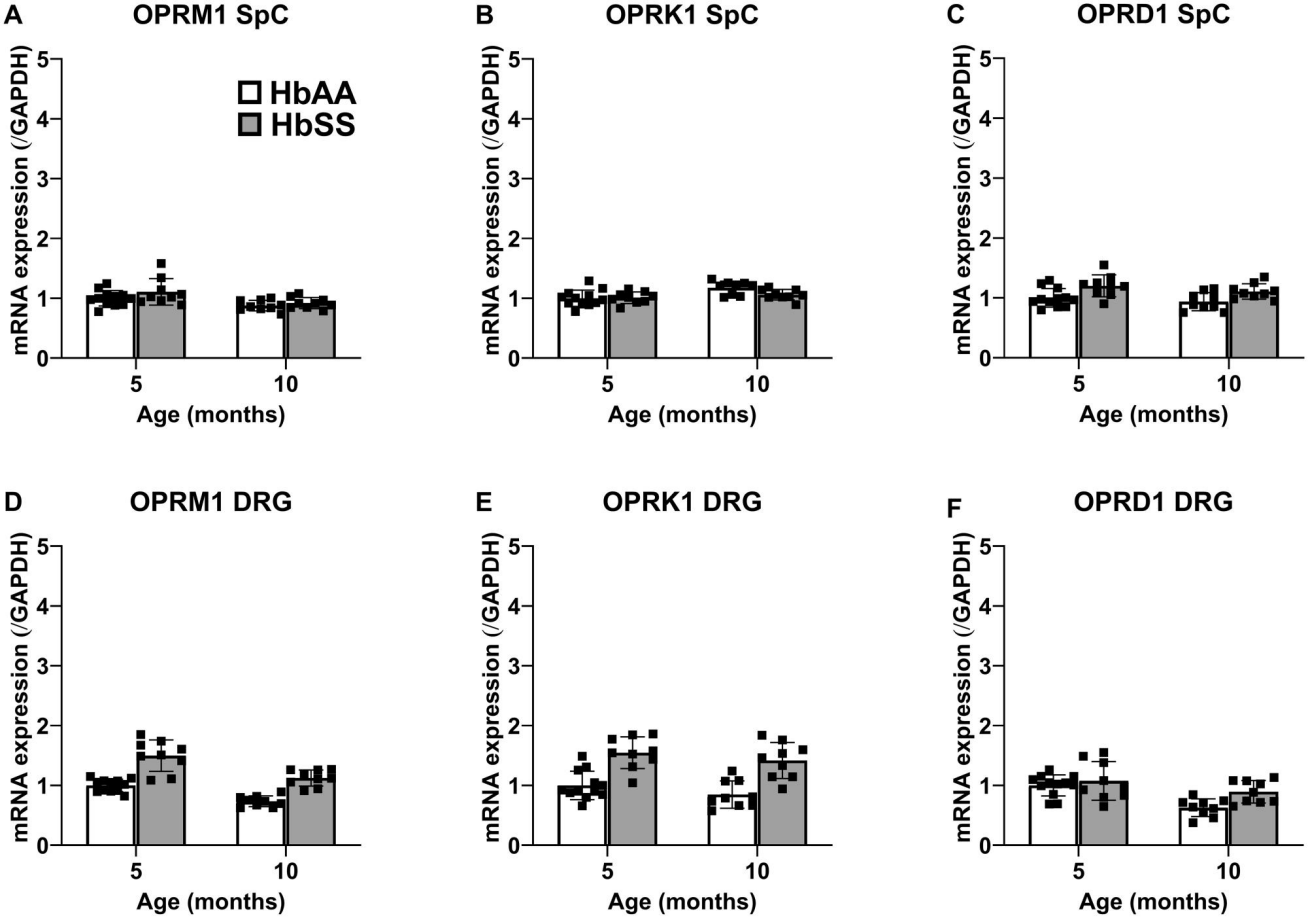
HbSS mice show changes in mRNA amounts of opioid receptors. (Panel A) 10-month HbSS mice show decreased amounts of spinal cord mu opioid receptor mRNA compared to 5-month HbSS mice. (Panel B) 10-month HbAA mice show increased kappa opioid receptor mRNA in spinal cord when compared to 5-month HbAA mice. (Panel C) HbSS mice spinal cords have increased delta opioid receptor mRNA when compared to age matched HbAA mice. (Panel D) HbSS DRGs show more mu opioid receptor mRNA than age matched HbAA mice. Additionally, 10-month mice show decreased amounts in both genotypes. (Panel E) More kappa opioid receptor mRNA was found in HbSS DRGs than in HbAA mice at both ages. (Panel F) 10-month HbSS DRGs had more delta opioid receptor mRNA than 10-month HbAA mice. However, 10-month HbAA mice had significantly decreased amounts compared to 5-month HbAA mice, with no difference between genotypes at 5 months. Bar graphs reflect mean ± SD from 7 to 12 mice/genotype/age. * *P* < .05; ** *P* < .01; *** *P* < .001 in age-matched HbSS mice vs HbAA mice. # *P* < .05; ## *P* < .01; ### *P* < .001 for 10 months vs 5 months. See Table S5 for two-way ANOVA for each dependent measure.

**Figure 8.**
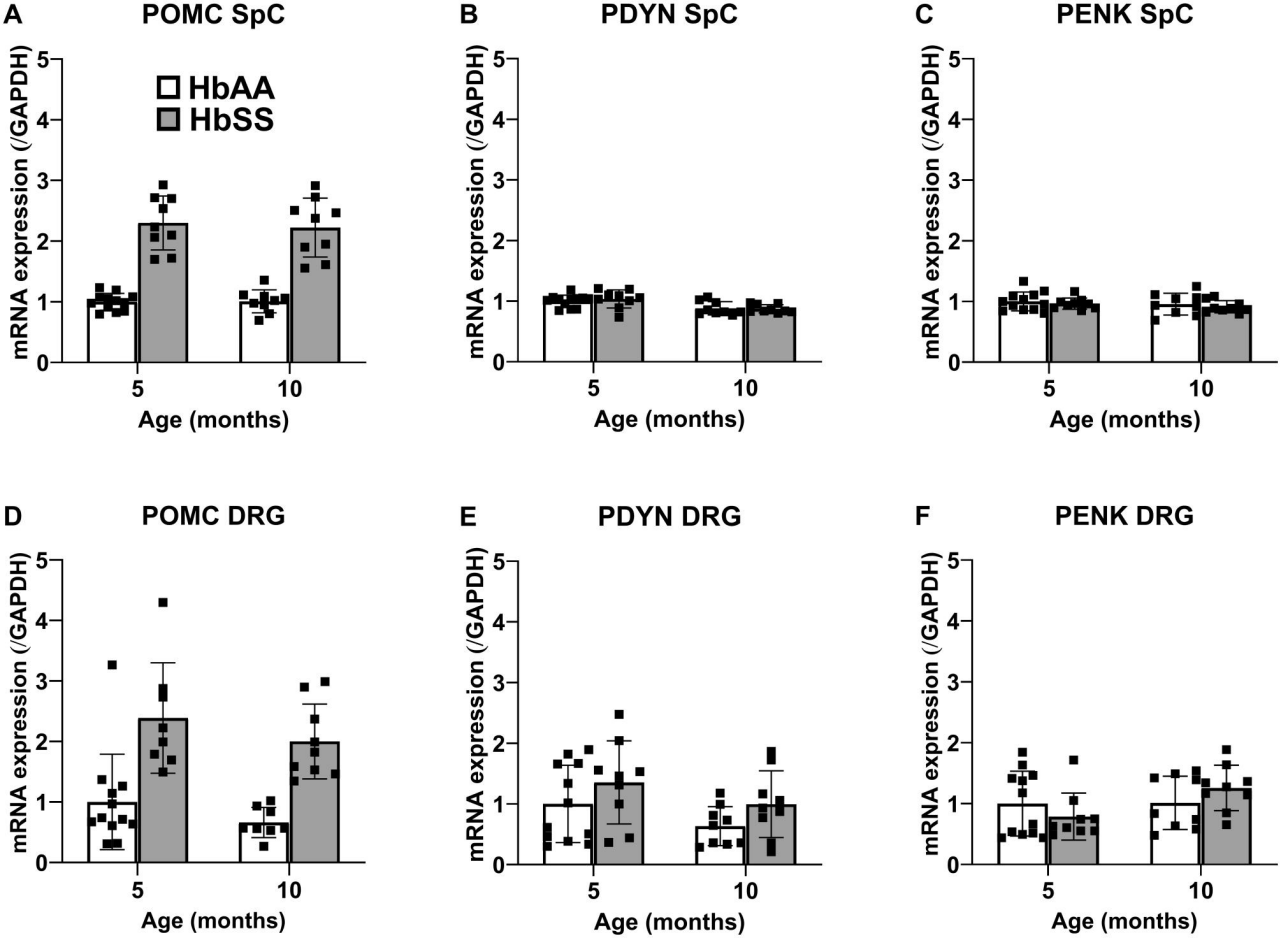
HbSS mice show age-dependent changes in mRNA amounts endogenous opioid precursors. (Panel A) In spinal cord, HbSS mice have higher POMC mRNA amounts than age matched HbAA mice. (Panel B) 10-month mouse spinal cords show less prodynorphin mRNA regardless of genotype. (Panel C) No change in proenkephalin mRNA between genotypes or ages in spinal cord. (Panel D) POMC mRNA amounts were ∼ 2-fold higher in HbSS mice at both 5 and 10 months of age. No changes were found, in DRGs, in either (Panel E) prodynorphin mRNA or (Panel F) proenkephalin mRNA between genotypes or ages. Bar graphs reflect mean +/- SD from 7 to 12 mice/genotype/age. * *P* < .05; ** *P*  < .01; *** *P*  < .001 in age-matched HbSS mice vs HbAA mice. # *P*  < .05; ## *P*  < .01; ### *P*  < .001 for 10 months vs 5 months. See Table S5 for two-way ANOVA for each dependent measure.

The mRNA data for opioid receptors are presented in Figure 7. While assorted main effects were found in spinal cord tissue, the magnitude of mRNA amounts differed very little among groups. In DRG, a significant main effect of genotype was found for mRNA for each opioid receptor with HbSS mice showing relatively large increases of OPRM1 and OPRK1 and minuscule increases in ORD1, irrespective of age. The quantitative mRNA data for precursors to endogenous opioid ligands are presented in Figure 8. POMC mRNA amounts in both spinal cord and DRG were markedly elevated in HbSS mice compared to HbAA control mice. However, genotype did not significantly affect relative amounts of PDYN and PENK mRNA.

**Figure 9.**
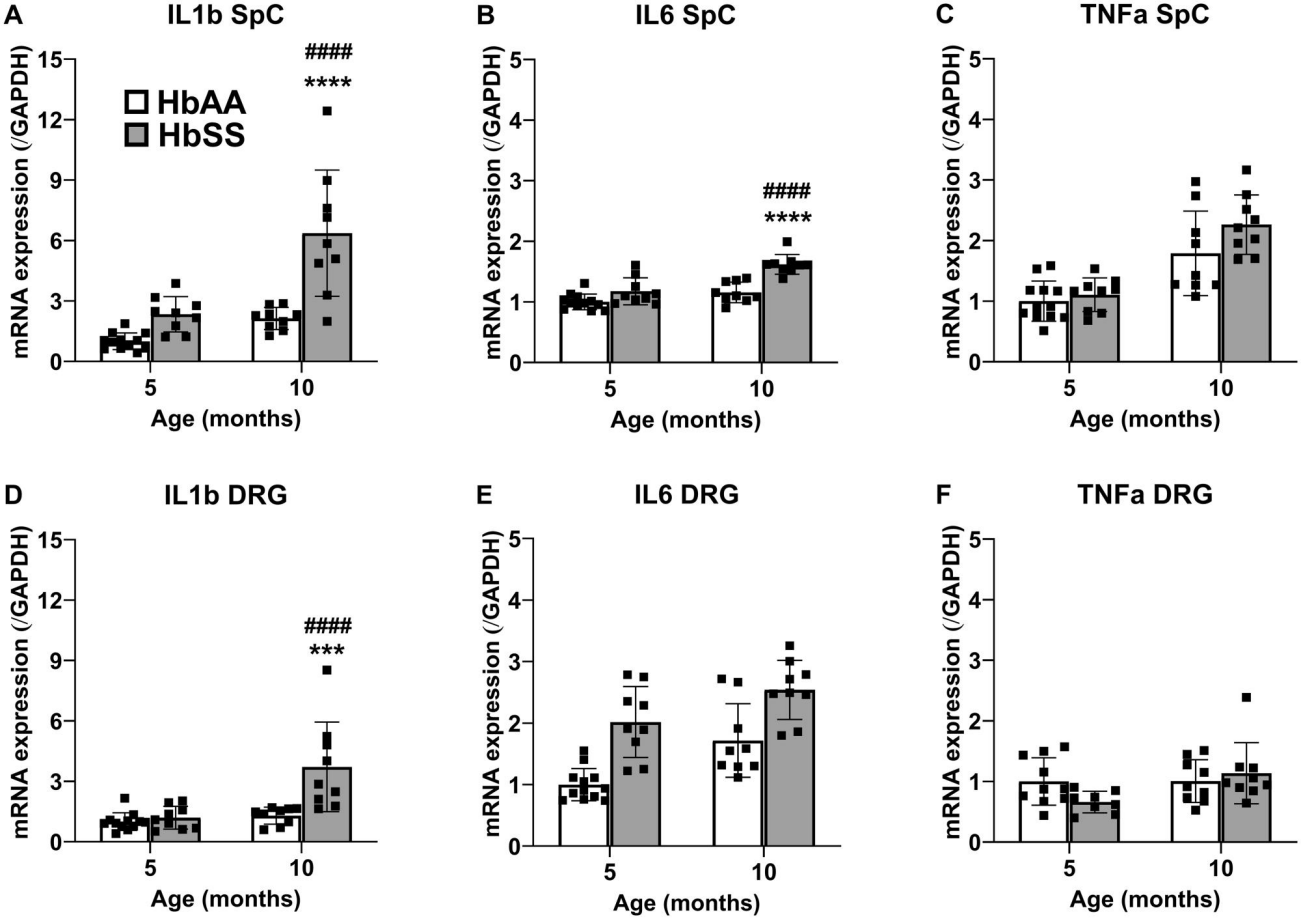
HbSS mice possess a distinct age-related pro-inflammatory cytokine mRNA profile. (Panel A) IL-1β mRNA is markedly increased in 10-month HbSS spinal cords. (Panel B) IL-6 mRNA is moderately increased in 10-month HbSS mice spinal cords. (Panel C) TFNα mRNA is significantly increased in 10-month spinal cords regardless of genotype. (Panel D) In DRG, IL-1β mRNA is sharply increased in 10-month HbSS mice. (Panel E) HbSS have moderately more IL-6 mRNA in DRG at 5- and 10-months when compared to HbAA controls, and 10-month animals produce more mRNA than 10-month animals of both genotypes. (Panel F) No changes were found in TFNα mRNA in DRG. Bar graphs reflect mean ± SD from 7 to 12 mice/genotype/age. * *P*  < .05; ** *P*  < .01; *** *P*  < .001 in age-matched HbSS mice vs HbAA mice. # *P*  < .05; ## *P*  < .01; ### *P*  < .001 for 10 vs 5 months. See Table S5 for two-way ANOVA for each dependent measure.

As shown in Figure 9, significant genotype × age interactions were found for IL-1β mRNA in spinal cord and DRG, with highest amounts detected in 10-month HbSS mice. Likewise, HbSS mice showed increased amounts of IL-6 mRNA compared with HbAA mice. A significant genotype by age interaction was found for IL-6 mRNA in spinal cord, while a main effect of genotype was found in DRG. Finally, genotype did not significantly affect TNFα mRNA, though older mice showed a significant increase in spinal cord.

## DISCUSSION

In these studies, we employed stimulus-evoked nociceptive assays, motor function behavioral paradigms, electrophysiological measures, and PCR to determine the contribution of age in the HbSS mouse model of SCD. We then tested oxycodone, a standard of care therapy, in ameliorating the SCD hyper-nociceptive phenotype. Although oxycodone produced antinociceptive effects in both genotypes, it failed to restore or worsened functional behavioral deficits. Our study provides a novel behavior testing paradigm that can be used to test established and novel pharmacological approaches on the SCD hyper-nociceptive phenotype.

Similar to other reports,^7^^,^^15^ we found that hypersensitivity to a cold stimulus emerged in 10-month HbSS mice. Likewise, others reported significant mechanical hypersensitivity only at 10 months.^7^ Although we found an overall genotype main effect in the von Frey assay, thresholds were significantly reduced as the mice aged. We also evaluated the contributions of genotype and age in various assays indicative of motor functional behavior. HbSS mice showed substantial age-related performance deficits in the nesting assay as well as general reductions in inverted screen, wheel running, burrowing and grip strength compared with HbAA control mice. Consistent with a previous report,^44^ HbSS mice showed deficits in voluntary wheel running. In contrast to Kohli et al,^7^ we did not observe age-related effects in grip strength. This is perhaps due to the use of mixed sexes in our study, though this factor did not significantly interact with age or genotype.

This study also found that HbSS mice showed lower amplitudes than HbAA mice, which may suggest poor axonal function and reduced myelin, respectively.^45^ Electrical stimulation studies have identified an age-related decrease in current thresholds in unmyelinated C fibers that corresponded to nocifensive vocalizations in HbSS mice.^13^ Likewise, the skin of HbSS mice shows age-dependent changes in nerve architecture (density and structural integrity).^7^ Collectively, these results are consistent with the possibility of physiological signs of peripheral neuropathy (ie, impaired peripheral nerve function) in HbSS mice.

A limitation of this study is that the secondary factor of sex was underpowered. Although sex differences were found in a variety of dependent measures, this factor did not alter HbSS phenotypic responses. Female mice had significantly reduced hotplate withdrawal latencies compared to male mice irrespective of genotype. In addition, grip strength was lower in female mice than in male mice. However, when grip strength was normalized to account for body mass, female mice showed better performance than male mice, and also showed improved performance in inverted screen and wheel running assays. Finally, 10-month-old female mice had had larger nerve conduction amplitudes than those of male mice, regardless of genotype and age. Sex hormones play a critical role in myelin development and myelin binding protein expression differs between sexes,^46^ which may account for the nerve conductance findings.

Because neuropathy is associated with neuronal hyperexcitability in other mouse neuropathic pain models (eg, spared nerve injury and chemotherapy-induced peripheral neuropathy^40^^,^^47^) and DRG neurons play an integral role in chronic pain,^43^ we assessed whether primary afferent (A-delta and C fibers) DRG neurons from HbSS mice show increased excitability compared to controls. DRG neurons from HbSS mice showed hyperexcitability. Specifically, HbSS mice required significantly less stimulus to elicit an action potential (ie, rheobase), and concomitantly had more action potentials at 2× Rheobase than HbAA mice. No differences were found in resting membrane potential. Due to logistical constraints, only 5-month female mice were used for the DRG electrophysiological experiment. Although the effects of sex and age on neuronal hyperexcitability from HbSS mice remain to be determined, these data complement the results of a study showing that DRGs from HbSS mice exhibited increased spontaneous activity, larger receptive fields, and prolonged after discharges to mechanical stimuli.^16^ Other groups have also identified profound activation of Ca^2+^/calmodulin-dependent protein kinase II (CaMKIIα) in DRG and spinal cord in HbSS-BERK mice, with inhibitors of this complex ameliorating SCD hyper-nociception.^48^ These ex vivo data support in vivo similar studies, which have identified nociceptor sensitization in anesthetized HbSS tibial nerve afferent fibers.^14^

Opioids, particularly morphine and oxycodone, are standard treatments for SCD pain, though their untoward side effects (eg, constipation, addiction, and respiratory depression) raise substantial concerns about their use for chronic pain conditions.^49^ Here, we tested oxycodone in 10-month HbSS and HbAA mice, an age showing the most severe hyper-nociceptive phenotype. Oxycodone ameliorated mechanical hypersensitivity and thermal hyperalgesia in HbSS mice, with antinociception occurring at higher doses. Similarly, oxycodone produced significant thermal antinociceptive effects and elevated von Frey paw thresholds in HbAA mice. In contrast, oxycodone worsened or failed to ameliorate deficits in the functional behavioral assays. Specifically, oxycodone disrupted performance of HbSS mice in the inverted screen and nesting assays, but did not affect grip strength, which was already substantially reduced compared to HbAA mice. Conversely, Kohli et al^7^ reported that morphine increased grip strength in HbSS mice. Collectively, these results indicate that while oxycodone produced antinociceptive effects, its impairment of functional behavior underscores its limitations for the treatment of chronic SCD pain. Further, the use of functional behavioral assays used here may prove a useful screening tool for future novel analgesics for the treatment of SCD pain, as well as other types of chronic pain syndromes. Improving screening methods in laboratory animal models, such as those described here, may help reduce attrition rates of candidate drugs in human clinical trials. Moreover, optimized preclinical screening models may improve efficacy and safety data before target molecules are transitioned to human studies, and increase the likelihood that novel analgesics improve overall quality of life in patients with chronic pain syndromes. As such, preclinical evaluation of potential pain pharmacotherapies for chronic SCD pain, should examine the consequences of repeated administration as well as employ stimulus-evoked behavior and motor functional assays. Future studies will be required to investigate whether oxycodone improves functional behavior in younger HbSS mice.

The mechanism by which SCD elicits hypersensitive nociceptive neurons associated with chronic pain remains unknown. Here, we procured DRG and spinal cord from 5- and 10-month-old HbSS and HbAA mice to quantify relative mRNA levels of biomarkers of interest pertaining to the endogenous opioid system and inflammatory cytokines. We found more mRNA for mu and kappa opioid receptor in DRG from HbSS mice compared to age-matched HbAA mice. Further, HbSS mice had significant increases of POMC mRNA amounts, the precursor to beta-endorphins, in both spinal cord and DRG. In contrast to the present results, other studies found no difference in relative gene expression of the proteins and molecules explored here (PDYN, PENK, and the mu, kappa, delta opioid receptors) between HbSS and HbAA 8-month-old mice^15^ or in the DRG of ∼2.5- and 5-month-old HbSS mice.^50^ Accordingly, further work is necessary to examine the influence of subtle procedural differences between the studies, as well as the potential of differences in the mixed background.

Inflammation is a widely characterized component of SCD.^51^^,^^52^ Widespread, chronic inflammation is both a byproduct of the hemolysis and ischemic damage of SCD, while also contributing to acute vaso-occlusive events and further damage to tissues, which may include sensory neurons.^53^ Further, SCD patients produce more pro-inflammatory mediators leading to the production of the inflammasome complex and the release of further pro-inflammatory cytokines, such as IL-1β.^54^ However, it should be noted that the procedures used in the present study do not model ischemia-induced acute episodes of SCD. Nonetheless, inflammation likely contributes to the development of the hyper-nociceptive phenotype in HbSS mice. Consistent with IL-1β as a target of interest in the pathogenesis and negative sequelae of SCD,^55^ we found that this cytokine mRNA was markedly elevated in spinal cord and DRG from HbSS mice, with a significant increase in 10-month mice compared to 5-month mice. Other studies have found that HbSS mice possessing elevated IL-1β show resistance to the analgesic effects of acupuncture.^56^ However, recent clinical trials targeting IL-1β failed to reduce SCD pain in human subjects, underscoring the complex mechanisms of SCD pain.^57^ HbSS mice also showed overall increases of IL-6 mRNA in DRG and age-related increases in spinal cord.

While it is plausible that these aforementioned mRNA changes in components of the endogenous opioid system or cytokines are a direct response to chronic nociception and/or contribute to the HbSS hyper-nociceptive phenotype, the cause remains unclear. The consequences of ischemia in SCD may be a contributing factor, though as indicated above the present study did not employ a model of ischemia-induced SCD. In addition, the lack of available tissue from 2-month-old mice available precluded a full assessment of age. Finally, it is important to note that the present results pertain to mRNA levels. Thus, further studies are needed to include a full age range of mice to ascertain whether genotype differences occur at the protein level, as well as assess whether alteration in binding or signal transduction occur.

The present study builds upon and extends previous work modeling the functional impairments of SCD in HbSS mice, and thus offers further utility of the HbSS mouse model in testing new therapeutic strategies in assays beyond those previously described,^7^^,^^58–60^ as well as potentially preventing age-related progression. Moreover, this study makes the unique observation that while acute oxycodone reversed the hyper-nociceptive phenotype in aged HbSS mice, it worsened or did not improve functional behavioral deficits, suggesting it may be suboptimal for restoring functional behaviors. Accordingly, this work provides a template to investigate established and novel analgesic strategies to treat chronic pain associated with SCD, as well as the accompanying functional deficits and inflammation.

## Supplementary Material

yoaf033_Supplementary_Data

## Data Availability

All data will be available upon request.
