# Supplementary material for: Behavioral and transcriptional effects of age in HbSS-BERK humanized SCD mice
Source: J Sick Cell Dis. 2025 Oct 7;2(1):yoaf033. doi: 10.1093/jscdis/yoaf033 (PMC12619645; doi:10.1093/jscdis/yoaf033)
Supplement: yoaf033_Supplementary_Data [file yoaf033_supplementary_data.docx]

**Supplementary Information**

**Behavioral and Transcriptional Effects of Age in HbSS-BERK Humanized Sickle Cell Disease Mice**

Kennedy N. Goldsborough, Ph.D.

Department of Pharmacology/Toxicology, Virginia Commonwealth University, Richmond, VA, USA

ORCID ID: 0000-0001-9403-2721

Michael W. Taylor, B.S.

Department of Pharmacology/Toxicology, Virginia Commonwealth University, Richmond, VA, USA

ORCID ID: 0009-0008-8400-4039

Bryan D. McKiver, Ph.D.

Department of Pharmacology/Toxicology, Virginia Commonwealth University, Richmond, VA, USA

ORCID ID: 0000-0002-1447-5803

Karan H. Muchhala, Ph.D.

Department of Pharmacology/Toxicology, Virginia Commonwealth University, Richmond, VA, USA

ORCID ID: 0000-0002-2594-1347

Molly E. Sonenklar, M.D.

Department of Pediatric Hematology/Oncology, Virginia Commonwealth University, Richmond, VA, USA

Now at: Division of Pediatric Hematology/Oncology, Louisiana State University Health Sciences Center, LA, USA

ORCID ID: 0009-0003-8923-8612

Atuahene Adu-Gyamfi, B.S.

Department of Pharmacology/Toxicology, Virginia Commonwealth University, Richmond, VA, USA

ORCID ID: 0000-0001-7921-8800

Sara M. Herz, B.S.

Department of Pharmacology/Toxicology, Virginia Commonwealth University, Richmond, VA, USA

ORCID ID: 0009-0004-8763-5670

Dawn K. Jessup, Ph.D.

Department of Pharmacology/Toxicology, Virginia Commonwealth University, Richmond, VA, USA

ORCID ID: 0000-0001-9104-7151

Joyce A. Lloyd, Ph.D.

Department of Human and Molecular Genetics, Virginia Commonwealth University, Richmond, VA, USA

ORCID ID: 0000-0003-0952-9923

Hamid I. Akbarali, Ph.D.

Department of Pharmacology/Toxicology, Virginia Commonwealth University, Richmond, VA, USA

ORCID ID: 0000-0003-1423-0774

M. Imad Damaj, Ph.D.

Department of Pharmacology/Toxicology, Virginia Commonwealth University, Richmond, VA, USA

ORCID ID: 0000-0002-8859-029X

Kalpna Gupta, Ph.D.

Division of Hematology/Oncology, Department of Medicine, University of California, Irvine, CA, USA

ORCID ID: 0000-0001-9381-9979

Wally R. Smith, M.D.

Department of Internal Medicine, Virginia Commonwealth University, Richmond, VA, USA

ORCID ID: 0000-0002-4122-5367

Aron H. Lichtman, Ph.D.

Department of Pharmacology/Toxicology, Virginia Commonwealth University, Richmond, VA, USA

ORCID ID: 0000-0001-5601-985X

Correspondence: Aron Lichtman, PhD, Virginia Commonwealth University, 1220 E Broad Street, Box​​ 980613, Richmond, VA; Phone: (804) 828-8480; Email: aron.lichtman@vcuhealth.org

**AUTHOR CONTRIBUTIONS**

Conceptualization: KG, AHL, WRS, JAL, MID; Investigation: KG, KM, SH, AA, MS, BM, DJ; Data Analysis: KG, KM, BM, MT; Writing: KG, MT, KM, JAL, AHL; Supervision: AHL, HA, MID; This paper was not composed by CHATgpt.

Manuscript category: Original article

Funding: This research was supported by The Central Virginia Center on Drug Abuse Research (5P30DA033934), NIH RO1HL147562, the IGNITE KUH Training Core (5TL1DK132771), Sickle Cell Disease Pain Analgesia And Integrative Network (1U24AT012868-01), the Southern Regional Education Board, a VCU Breakthrough Grant, VCU School of Pharmacy Start Up funds, VCU School of Medicine Bridge Funds, and the Children’s Health Research Institute Fellows Research Program at the Children’s Hospital of Richmond at VCU.

Conflicts of interest: Kalpna Gupta: Honoraria: *Tautona Group*, *Novartis* and *CSL Behring*. Research Grants: *Cyclerion*, *1910 Genetics, Novartis*, *Grifols*, *UCI Foundation and SCIRE Foundation.*

Data availability statement: All data will be available upon request.

**Statistical Analyses**

#### **Supplementary Table 1.** [**Statistical Analyses: Stimulus-evoked Behaviors (Figure 1)**](https://www.zotero.org/google-docs/?p87IQ3)**.**

| [**Assay**](https://www.zotero.org/google-docs/?p87IQ3) | [**Sample Size**](https://www.zotero.org/google-docs/?p87IQ3) | **Two-way ANOVA** |
| --- | --- | --- |
| von Frey | [N = 8/genotype/age](https://www.zotero.org/google-docs/?p87IQ3) | Age: F (2, 42) = 6.30, P < 0.01  Genotype: F (1, 42) = 95.71 P < 0.0001  [Interaction: F(2, 42) < 0.05; P = 0.86](https://www.zotero.org/google-docs/?p87IQ3) |
| [Hot](https://www.zotero.org/google-docs/?p87IQ3)plate | [N = 6/genotype/age](https://www.zotero.org/google-docs/?p87IQ3) | Age: F (2, 30) = 3.9, p < 0.05  Genotype: F (1, 30) = 88.30, p < 0.0001  Interaction: F (1, 30) = 0.98, p = 0.386 |
| [Acetone](https://www.zotero.org/google-docs/?p87IQ3) | [N = 6/genotype/age](https://www.zotero.org/google-docs/?p87IQ3) | Age: F (2, 30) = 49.2, p < 0.0001  Genotype: F (1, 30) = 66.62, p < 0.0001  Interaction: F (1, 30) =18.48, p < 0.0001 |

####

#### [**Supplementary Table 2. Statistical Analyses: Non-evoked Motor Functional Behaviors (Figure 2)**](https://www.zotero.org/google-docs/?p87IQ3)**.**

| [**Assay**](https://www.zotero.org/google-docs/?p87IQ3) | [**Sample Size**](https://www.zotero.org/google-docs/?p87IQ3) | [**Two-way ANOVA**](https://www.zotero.org/google-docs/?p87IQ3) |
| --- | --- | --- |
| [Grip](https://www.zotero.org/google-docs/?p87IQ3) Strength | N = 7-9/genotype/age | Age: F (2, 42) = 0.2276, p = 0.797  Genotype: F (1, 42) = 24.09, p < 0.0001  Interaction: F (1, 42) = 1.469, p = 0.242 |
| Inverted Screen | N = 8/genotype/age | Age: F (2, 42): 3.977, p < 0.05  Genotype: F (1, 42) =44.41, p < 0.0001  Interaction: F (1, 30) = 0.154, p = 0.858 |
| Wheel Running | N = 7-9/genotype/age | Age: F (2, 38): 3.43, p < 0.05  Genotype: F (1, 38) =13.1, p < 0.0001  Interaction: F (1, 38) =0.247, p = 0.782 |
| [Burrowing](https://www.zotero.org/google-docs/?p87IQ3) | N = 7-9/genotype/age | Age: F (2, 40) = 0.996, p = 0.378  Genotype: F (1, 40) = 28.3, p < 0.0001  Interaction: F (1, 40) =0.505, p = 0.607 |
| [Nesting (Consolidation](https://www.zotero.org/google-docs/?p87IQ3)) | N = 13-16/genotype/age | Age: F (2, 73) = 3.12, p<0.05  Genotype: F (1, 73) = 47.5, p < 0.0001  Interaction: F (1, 73) = 5.73, p < 0.01 |

####

#### **[Supplementary Table 3. Statistical Analyses: Oxycodone Data (Figures 5 and 6). Sample size: n = 7-8 mice/genotype/age.](https://www.zotero.org/google-docs/?p87IQ3)**

| [**Assay**](https://www.zotero.org/google-docs/?p87IQ3) | [**Two-Way (Repeated Measures) ANOVA**](https://www.zotero.org/google-docs/?p87IQ3) |
| --- | --- |
| [von Frey](https://www.zotero.org/google-docs/?p87IQ3) | Genotype: F(1, 14) = 105.8, p < 0.0001  Dose: F(4, 56) = 32.82, p < 0.0001  Genotype x Dose: F(4,56) = 0.56, p = 0.69 |
| [Hotplate](https://www.zotero.org/google-docs/?p87IQ3) | Genotype: F(1, 14) = 64.26, p < 0.0001  Dose: F(4, 56) = 95.26, p < 0.0001  Genotype x Dose: F(4, 56) = 3.06, p < 0.05 |
| [Grip Strength](https://www.zotero.org/google-docs/?p87IQ3) | Genotype: F(1, 13) = 11.9, p<0.01  Dose: F(4, 52) = 6.17, p < 0.01  Genotype x Dose: F(4, 52) = 2.60, p < 0.05 |
| [Inverted Screen](https://www.zotero.org/google-docs/?p87IQ3) | Genotype: F(1, 13) = 7.53, p < 0.05  Dose: F(4, 52) = 1.58, p = 0.19  Genotype x Dose: F(4, 52) = 2.91, p < 0.05 |
| [Nesting (Consolidation](https://www.zotero.org/google-docs/?p87IQ3)) | Genotype: F (1, 13) = 0.83, p = 0.38  Dose: F(2, 26) =18.2, p < 0.0001  Genotype x Dose: F(2, 26) = 3.30, p = 0.053 |

#### [**Supplementary Table 4. Statistical Analyses: Opioid mRNA Data (Figures 7 and 8)**](https://www.zotero.org/google-docs/?p87IQ3)**. Sample sizes: 5-month-old HbAA mice (n = 12), 5-month-old HbSS (n = 9), 10-month-old HbAA mice (n = 9), and 10-month-old HbSS (n = 9).**

| [**Gene**](https://www.zotero.org/google-docs/?p87IQ3) | **Two-way ANOVA (Spinal Cord)** | **Two-way ANOVA (DRG)** |
| --- | --- | --- |
| [OPRM1](https://www.zotero.org/google-docs/?p87IQ3) | Genotype: F(1, 35) = 2.65, p = 0.11  Age: F(1, 35) = 11.5, p < 0.01  Genotype x Dose: F(1,35) = 0.50, p = 0.48 | Genotype: F(1, 35) = 75.1, p < 0.0001  Age: F(1, 35) = 39.2, p < 0.0001  Genotype x Dose: F(1,35) = 1.13, p = 0.30 |
| OPRK1 | Genotype: F(1, 35) = 2.2, p = 0.14  Age: F(1, 35) = 9.8, p < 0.05  Genotype x Dose: F(1,35) = 3.0, p = 0.09 | Genotype: F(1, 35) = 45.0, p < 0.0001  Age: F(1, 35) = 2.88, p = 0.10  Genotype x Dose: F(1,35) = 0.02, p = 0.89 |
| [ORD1](https://www.zotero.org/google-docs/?p87IQ3) | Genotype: F(1, 35) = 13.7, p < 0.001  Age: F(1, 35) = 2.4, p = 0.13  Genotype x Dose: F(1,35) = 0.089, p = 0.77 | Genotype: F(1, 35) = 6.0, p < 0.05  Age: F(1, 35) = 15.8, p < 0.001  Genotype x Dose: F(1,35) = 1.87, p = 0.18 |
| [POMC](https://www.zotero.org/google-docs/?p87IQ3) | Genotype: F(1, 35) = 134, p < 0.0001  Age: F(1, 35) = 0.1, p = 0.75  Genotype x Dose: F(1,35) = 0.15, p = 0.70 | Genotype: F(1, 33) = 34.2, p < 0.0001  Dose: F(1, 33) =2.4, p = 0.13  Genotype x Dose: F(1, 33) = 0.01, p= 0.92 |
| PDYN | Genotype: F(1, 35) = 0.16, p = 0.69  Age: F(1, 35) = 15.7, p < 0.001  Genotype x Dose: F(1,35) = 0.38, p = 0.54 | Genotype: F(1, 35) = 3.76, p = 0.06  Age: F(1, 35) = 3.85, p = 0.06  Genotype x Dose: F(1,35) = 0.00, p = 0.98 |
| PENR | Genotype: F(1, 35) = 0.87, p = 0.39  Age: F(1, 35) = 1.2, p = 0.29  Genotype x Dose: F(1,35) = 0.006, p = 0.94 | Genotype: F(1, 35) = 0.012, p = 091  Age: F(1, 35) = 2.81, p = 0.10  Genotype x Dose: F(1,35) = 2.52, p = 0.12 |

#### [**Supplementary Table 5. Statistical Analyses: Cytokine mRNA Data (Figure 9)**](https://www.zotero.org/google-docs/?p87IQ3)**. Sample sizes: 5-month-old HbAA mice (n = 12), 5-month-old HbSS (n = 9), 10-month-old HbAA mice (n = 9), and 10-month-old HbSS (n = 9).**

| [**Gene**](https://www.zotero.org/google-docs/?p87IQ3) | **Two-way ANOVA (Spinal Cord)** | **Two-way ANOVA (DRG)** |
| --- | --- | --- |
| [IL1β](https://www.zotero.org/google-docs/?p87IQ3) | Genotype: F(1, 35) = 29.3, p < 0.0001  Age: F(1, 35) = 25.1, p < 0.0001  Genotype x Dose: F(1,35) = 7.88, p < 0.01 | Genotype: F(1, 35) = 12.6, p < 0.01  Age: F(1, 35) = 14.6, p < 0.001  Genotype x Dose: F(1,35) = 9.12, p < 0.01 |
| IL6 | Genotype: F(1, 35) = 33.2 p < 0.0001  Age: F(1, 35) = 29.5, p < 0.0001  Genotype x Dose: F(1,35) = 6.8, p < 0.05 | Genotype: F(1, 35) = 35.0, p < 0.0001  Age: F(1, 35) = 15.9, p < 0.001  Genotype x Dose: F(1,35) = 0.4, p = 0.53 |
| [TNFα](https://www.zotero.org/google-docs/?p87IQ3) | Genotype: F(1, 35) = 3.68, p = 0.06  Age: F(1, 35) = 41.7, p < 0.0001  Genotype x Dose: F(1,35) = 1.48, p = 0.23 | Genotype: F(1, 32) = 0.68, p = 0.42  Age: F(1, 32) = 3.57, p = 0.07  Genotype x Dose: F(1,32) = 3.40, p = 0.07 |
